# Meeting 24-hour movement guidelines and the relationship with active commuting to school in Spanish urban areas

**DOI:** 10.3389/fspor.2025.1588118

**Published:** 2025-05-22

**Authors:** Evelyn Martín-Moraleda, Iván Pinilla-Quintana, Cristina Romero-Blanco, Antonio Hernández-Martínez, Fabio Jiménez-Zazo, Alberto Dorado-Suárez, Virginia García-Coll, Esther Cabanillas-Cruz, María Teresa Martínez-Romero, Manuel Herrador-Colmenero, Ana Queralt, Nuria Castro-Lemus, Guy Faulkner, Susana Aznar

**Affiliations:** ^1^ PAFS Research Group, Faculty of Sports Sciences, University of Castilla-La Mancha, Toledo, Spain; ^2^ PAFS Research Group, Faculty of Nursing, University of Castilla-La Mancha, Ciudad Real, Spain; ^3^ Postdoctoral Fellow “Margarita Salas”, University of Murcia, Murcia, Spain; ^4^ PROFITH “PROmoting FITness and Health through Physical Activity” Research Group, Sport and Health University Research Institute (iMUDS), Department of Physical Education and Sports, Faculty of Sport Sciences, University of Granada, Granada, Spain; ^5^ “La Inmaculada” Teacher Training Centre, University of Granada, Granada, Spain; ^6^ AFIPS Research Group, Department of Nursing, University of Valencia, Valencia, Spain; ^7^ FENIX Research Group, Faculty of Sports Sciences, University of Sevilla, Sevilla, Spain; ^8^ POP-PA Lab, School of Kinesiology, Faculty of Education, University of British Columbia, Vancouver, BC, Canada

**Keywords:** active commuting to school, physical activity, sedentary behavior, sleep, adolescents, lifestyle

## Abstract

**Introduction:**

New guidelines from Canada indicate that the integration of all movement behaviours [physical activity (PA), screen time (ST) and sleep time (SLT)] is important for health across the day. Active commuting to school (ACS) provides an opportunity to increase PA levels daily while replacing sedentary time passively commuting. The aim of this study was to assess the prevalence of ACS, and if meeting the different guidelines included in the 24-Hour Movement Guidelines is associated with ACS in adolescents.

**Methods:**

A cross-sectional study was conducted to investigate adherence to ACS in relation to environment, psychosocial variables, and healthy lifestyle factors in Spanish adolescents. The final sample included 686 adolescents (mean age = 14.81 ± 0.52 years, 50.8% girls) from seven Spanish urban areas stratified by SES and walkability. Adolescents filled in an “*ad hoc*” questionnaire on the mode of commuting to school and lifestyle behaviors based on the PACO&PACA questionnaire. Logistic regression models were fit to evaluate if meeting the 24-Hour Movement Guidelines was associated with ACS. The level of significance was set at *p* ≤ 0.05.

**Results:**

A total of 55.8% adolescents commuted actively from home to school. Data showed active commuters (AC) met 24-Hour Movement Guidelines more than passive commuters (PC). Evidence from the binary logistic regression indicated that those who met two Guidelines (aOR = 1.62; 95% CI: 1.01–2.59; *p* = 0.047) had higher odds of ACS than those who did not meet any 24-Hour Movement Guidelines. In particular, girls who met two or all 24-Hour Movement Guidelines had higher odds of ACS than those who met none (OR = 2.06, 95% CI: 1.10–3.87; OR = 2.30, 95% CI: 1.00–5.27, respectively).

**Conclusions:**

In conclusion, our study suggests that meeting more 24-Hour Movement Guidelines is associated with ACS in adolescents. In addition, promoting ACS as a strategy to meet the 24-Hour Movement Guidelines is recommended particularly for adolescent girls.

## 1 Introduction

It is known that physical activity (PA), sleep time (SLT) and sedentary behaviors (e.g., screen time, ST) are associated with adolescents’ health (1–4). To date, most research has examined these behaviors in isolation, but recent trends propose to investigate several lifestyle behaviors together as they may have greater benefits than their individual impacts (5). In fact, the World Health Organisation (WHO) is preparing global combined movement guidelines for children and adolescents in accordance with the WHO Global Action Plan on Physical Activity (GAPPA) 2018–2030 (6).

Specifically, new 24-Hour Movement Guidelines from Canada, indicate that the integration of all movement-related behaviours (PA, SLT, and ST) is important for health (7). These guidelines recommend that children and adolescents should accumulate at least 60 min per day of moderate-to-vigorous PA, involving a variety of aerobic activities, but also they should accumulate 9–11 h of sleep per day (for 5–13 years old) or 8–10 h of sleep per day (for 14–17 years old); and they should have low levels of sedentary behaviors, this includes, less than 2 h/day of recreational ST. A key recommendation is ultimately that preserving sufficient sleep, trading indoor time for outdoor time, and replacing sedentary behaviours and light physical activity with additional moderate to vigorous PA can provide greater health benefits (8).

Meeting the 24-Hour Movement Guidelines has been associated with better body composition, cardiorespiratory and musculoskeletal fitness, academic achievement and cognition, emotional regulation, pro-social behaviours, cardiovascular and metabolic health, and overall quality of life (7, 8). Despite the health benefits of meeting these guidelines, a recent meta-analysis showed that most young people from 23 countries failed to meet the three components of the 24-Hour Movement Guidelines, particularly adolescents and girls (9). Therefore, these results highlighted the need to develop age-and sex-specific strategies to promote these movement-related behaviors from the early stages of life (9–11).

Active commuting to school (ACS) provides an opportunity to increase adolescents’ PA levels daily (12, 13) and it could help in the achievement of the 24-Hour Movement Guidelines. Systematic reviews demonstrate that children and youth who actively commute to/from school are more physically active than those who do not (14). Replacing a sedentary behaviour (e.g., driving by car) with one that is physically active (e.g., cycling to school) is in line with the underlying 24-Hour Movement Guidelines. Sleep duration might also be potentially impacted given the potential time demand differences of active vs. passive commuting to school (e.g., waking up earlier to allow time to actively commute to school). One study analyzed associations among ACS with the different components of the 24-Hour Movement Guidelines independently, but not all the 24-Hour Movement Guidelines together (15). That study showed one individual relationship between ACS with SLT but without significant differences between ACS and ST. However, the relationship between ACS and 24-Hour Movement Guidelines has not been analyzed in adolescents. This may be an interesting focus given the inter-relationship between these behaviours. But, there is scarced research in the combined adherence of the 24-Hour Movement Guidelines and its association with ACS.

Therefore, the aim of this study was (1) to assess the relationship between ACS and the meeting of the different guidelines included into the 24-Hour Movement Guidelines in Spanish adolescents and (2) to assess if this relationship differs by gender. This former analysis could help us in increasing the understanding in behavioral, developmental, or psychosocial factors that differ between boys and girls in adolescence.

## 2 Materials and methods

### 2.1 Study design

A cross-sectional study was conducted to investigate adherence to ACS in relation to environment, psychosocial variables, and healthy lifestyle factors in Spanish adolescents. The study design, protocols and methodology of the PACO&PACA Research Project were approved by the Ethics Committee for Research with Drugs-SESCAM (ID: C-392) and in accordance with the Helsinki Declaration (1961) revised in Fortaleza (2013). The study protocol has already been published (16).

### 2.2 Participants and recruitment

The current study captured a sample of adolescents from seven Spanish urban areas (Toledo, Cuenca, Albacete, Puertollano, Granada, Sevilla, and Valencia).

To ensure representativeness, according to the walkability and socioeconomic status (SES) scenarios, all schools from each city were classified according to SES and walkability in their census blocks prior to randomly selecting them. School census blocks were used as the smallest administrative areas, where SES was obtained from the average income of the census blocks was obtained from the National Institute of Statistics (INE) 2016. After, using Geographical Information System, walkability was calculated for each school included in the study and they were classified as low walkability or high walkability. From this classification, school areas were categorized as having low or high SES, and low or high walkability index. Schools’ environments were then classified according the four possible SES-Walkability combinations (High-High, High-Low, Low-Low, Low-High). This classification has already been done by previous research (17–20).

Assuming that 50% of the subjects in the population have the factor of interest (ACS), and an expected response rate of 90%, the study would require a sample size of 667 for estimating the expected proportion with 4% absolute precision and 95% confidence (21).

Initial meetings were conducted with the staff of the schools to communicate the information about the research project. After this, adolescents and their parents/guardians and physical education teachers were fully informed verbally and in writing about the nature and purpose of the study. All parents/guardians signed an informed consent form prior to participation.

Adolescents were tested in a session during class time in each Spanish urban area. An experienced researcher first introduced the online questionnaire to students, explained the procedure for completing the survey and personally answered all participants’ questions. Adolescents answered the online questionnaire in the school about the mode of commuting to school and lifestyle behaviors.

For adolescents to be included in the study, the inclusion criteria were: (a) being in the 3rd grade of secondary school, (b) returning the signed consent form before the start of the study (c) being present on the day of data collection, and (d) having complete data for the variables used in this study. The initial sample consisted of 713 adolescents in the 3rd level of secondary school. Twenty-seven adolescents were excluded based on the inclusion criteria “d”. The final sample included 686 adolescents (mean age = 14.81 ± 0.52 years, 50.8% girls) meeting the inclusion criteria.

### 2.3 Instrument

For this study, all participants filled in an “*ad hoc*” questionnaire (PACO&PACA Questionnaire) (16) to measure their mode of commuting to school and their lifestyle behaviours. All scales included in the questionnaire were psychometrically studied previously.

### 2.4 Measures

#### 2.4.1 Mode of commuting to school

The *Mode and Frequency of Commuting To and From School Questionnaire* from the PACO Study was used, which has been previously validated with an accelerometer in Spanish children and adolescents (22) and is a feasible and reliable questionnaire (23).

It included the following question: “Think about the last 5 days you have attended school (not including today). Answer the following question: How did you go to school?” Possible options for each day were: I didn't go to school, walk, cycle, electric cycle, skateboard, kicking scooter, electric scooter, car, motorcycle, school bus, public bus, metro/train/tram/taxi/, and others. This item was dichotomized as “active commuting” based on reported walking or cycling to school as the usual mode, or passive commuting (rest of transports).

#### 2.4.2 The 24-hour movement guidelines

##### 2.4.2.1 Screen time

Adolescents provided data on their ST spent in four different devices during weekdays: mobile phone, television, computer, or console games. It was evaluated using the Screen-Time Sedentary Behavior Questionnaire (24) from the Healthy Lifestyle in Europe by Nutrition in Adolescence (HELENA) (25). Each adolescent selected one of the following categories/day per each device: (a) none, (b) less than half an hour, (c) between half an hour to an hour, (d) between one and two hours, (e) between two and three hours, (f) more than three hours. To unify ST, it was assigned an average minutes score to each possible answer (i.e., adolescents who spent 0 h or minutes scored 0 min, those who spent less than 30 min scored 15 min, between 30 min and 1 h scored 45 min, between 1 h and 2 h scored 90 min, between 2 h and 3 h scored 150 min and finally, adolescents who spent more than 3 h scored 180 (24). A new variable was created for this purpose, including the sum of the minutes from the four screen behaviours measured.

##### 2.4.2.2 Sleep time

Behaviours related to sleep were gathered by questions about the time when adolescents went to sleep, and the time when they woke up from the PASOS (Physical Activity, Sedentarism and Obesity in Spanish Youth) Study Questionnaire (26). They self-reported the exact time when they went to sleep and when they got up on a usual school day, and sleep duration was calculated on this basis. Adolescents were categorised as having “adequate sleep duration” when they met sleep time recommended by the Sleep Foundation, and “non-adequate sleep duration” when they did not meet the recommendations. According to the recommendations for young and old adolescents it was from 8 to 10 h (27).

##### 2.4.2.3 Physical activity

Adolescents’ PA was estimated by their Stages of Change (SoC) for their practice of PA using the scale of Marcus et al. (28). One of the theoretical models that has been useful in understanding and predicting PA behaviour change is the Transtheoretical Model (TTM). The TTM includes a temporal dimension of change, in which behaviour change is described as a process that takes place over time with a sequence of the stages through which individuals progress toward an adoption of regular behaviour. TTM was first focused on smoking cessation studies (29), but from the 1990s researchers started using the TTM in healthy lifestyle promotion, including PA, but mainly among adult populations (30). To study the process of adopting PA behaviour through the SoC in children and adolescence, is of great importance due to their well-documented decline in PA levels (31). The SoC in children and adolescents has shown a good internal and external validity for exercise behaviour when used in a sample of college students (32) so, the use of PA SoC in this study can contribute to improve the understanding of the physical activity-related behaviour.

This scale consisted of six mutually exclusive items for each of the six stages: pre-contemplation, contemplation, preparation, action, maintenance and, relapse. Examples are items for the pre-contemplation stage: “I currently do not exercise, and I do not intend to start exercising in the next 6 months” and maintenance stage: “I currently exercise regularly and have done so for longer than 6 months”. The relapse stage was measured by the following item: “Until recently, I have been doing exercise in my leisure time, but I have stopped doing it”. Response options for each item were “true” and “false”. Participants were divided into two groups based on their stage of change considering previous research in line with meeting PA guidelines (33–35): inactive (i.e., pre-contemplation, contemplation, and preparation) and active (i.e., action and maintenance).

### 2.5 Statistical analysis

Descriptive characteristics were presented as adjusted means with standard deviation for quantitative variables or as a proportion for categorical variables.

Chi-square statistical analysis were performed to evaluate differences between categorical variables. The proportion of active commuters (AC) and passive commuters (PC) who met the 24-Hour Movement Guidelines were determined.

Finally, binary logistic regression analysis were fit to assess the association of meeting the 24-Hour Movement Guidelines with ACS. A multivariate analysis was applied controlling for gender. The Odds Ratio (OR) and adjusted Odss Ratio (aOR) were estimated with a Confidence Interval (CI) of 95%.

The analysis was conducted using SPSS, IBM v. 25.0 for Windows. The level of significance was set at *p* ≤ 0.05, since in practice the *p*-value may be equal to 0.05 due to rounding or computational precision, excluding that value could lead to inconsistent decisions. This approach is also supported in widely used biostatistics texts (36).

## 3 Results

The final sample included 686 adolescents (mean age = 14.81 ± 0.52 years, 50.8% girls) meeting the inclusion criteria. Descriptive sample characteristics are presented in Table 1. A total of 55.8% adolescents commuted actively from home to school. When we compare the amount of achievement of the Canadian 24-Hour Movement Guidelines, only 8.9% met all the Guidelines, 33.4% met two, 42% met one, and 15.7% met none. In the whole sample, ST was the least achieved guideline followed by SLT and PA (16.9%, 56.3% and 62.2%, respectively).

**Table 1:** Sample characteristics.

Variable	*N* (%) *N* = 686	Mean (DE)
Age (years) mean (SD)		14.81 (0.52)
Sex		
Boys	337 (49.2)	
Girls	348 (50.8)	
24-hour movement guidelines		
Meet 0 guidelines	108 (15.7)	
Meet 1 guideline	288 (42)	
Meet 2 guidelines	229 (33.4)	
Meet 3 guidelines	61 (8.9)	
Screen time ≤120 min/day		
No	570 (83.1)	
Yes	116 (16.9)	
PA ≥60 min/day		
No	259 (37.8)	
Yes	427 (62.2)	
Sleep time ≥8 h		
No	300 (43.7)	
Yes	386 (56.3)	
Healthy behavior combinations		
None	108 (15.7)	
Only meet PA	168 (24.5)	
Only meet ST	8 (1.2)	
Only meet SLT	112 (16.3)	
PA + SLT	182 (26.5)	
SLT + ST	31 (4.5)	
ST + PA	16 (2.4)	
All	61 (8.9)	
Mode of transport		
Active	383 (55.8)	
Passive	303 (44.2)	

**Figure 1:**
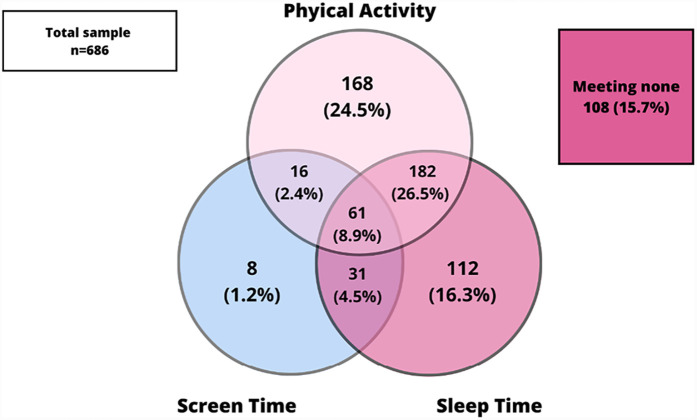
Meeting 24-hour movement guidelines in the total sample.

**Figure 2:**
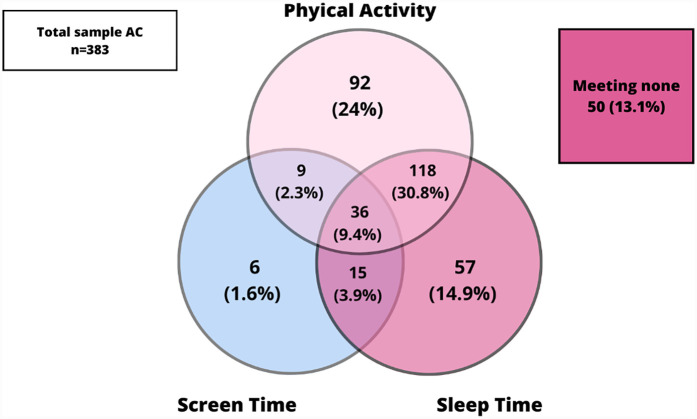
Meeting 24-hour movement guidelines in the AC sample.

**Figure 3:**
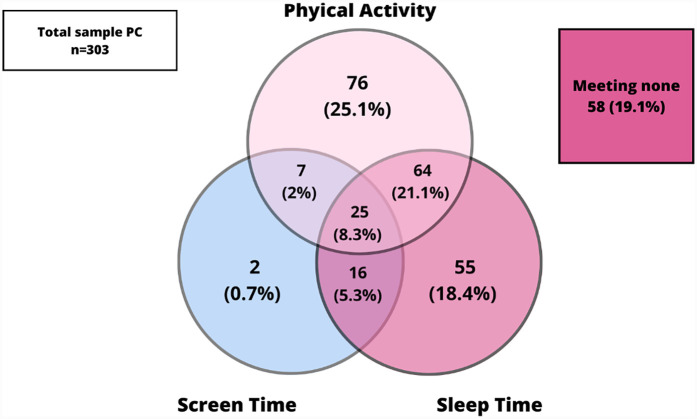
Meeting 24-hour movement guidelines in the PC sample.

Figure 1 shows the prevalence of adherence of the combined movement guidelines and its specific combinations (PA, SLT and ST) in the whole sample and, Figures 2, 3 presents the same prevalence of adherence but in this case according to AC vs. PC. If adolescents met two guidelines, the most frequent combination was meeting PA and SLT guidelines. When comparing this pattern of adherence to the combined movement guidelines according to mode of commuting to school, AC presented higher percentages of adolescents meeting two (specially PA + SLT) and also all three 24-Hour Movement Guidelines, than did PC. But, there were no significant differences in these previous results (*p* = 0.065).

Results from the binary logistic regression are presented in Tables 2, 3. Table 2 showed the associations between ACS, gender and meeting none, one, two or three 24-Hour Movement Guidelines in the total sample. AC achieved significantly more 24-Hour Movement Guidelines than PC (Table 2). Compared to meeting no guidelines, higher odds of ACS were found for those participants who met two guidelines (OR = 1.89; 95% CI: 1.19–3.01; *p* = 0.007) (Table 2). This associations remained after adjusting by gender, where participant who met two guidelines had higher odds of ACS than those who met none (aOR = 1.62; 95% CI: 1.01–2.59; *p* = 0.047) (Table 2). In terms of gender (Table 3), girls who met two or all 24-Hour Movement Guidelines had higher odds of ACS than those who met none (OR = 2.06; 95% CI: 1.10–3.87; OR = 2.30; 95% CI: 1.00–5.27, respectively). No significative evidence was found for boys (Table 3).

**Table 2:** Factors associated with mode of transport to school in a sample of adolescents.

Variable	Mode of transport		Bivariate analysis		Multivariate analysis	
	AC *n* (%) *N* = 383	PC *n* (%) *N* = 303	OR 95% CI	*P*-value	aOR 95% CI	*P*-value
Sex				**0.014**		**0.043**
Boys	209 (54.6)	130 (42.8)	**1**		**1**	
Girls	174 (45.4)	174 (57.2)	**1.45** **(1.08–1.95)**		**1.38** **(1.01–1.87)**	
24-hour movement guidelines				**0.042**		0.246
Meet 0 guidelines	50 (13.1)	58 (19.1)	1		1	
Meet 1 guideline	155 (40.5)	133 (43.9)	1.35 (0.87–2.11)	0.183	1.30 (0.83–2.04)	0.253
Meet 2 guidelines	142 (37.1)	87 (28.7)	**1.89** **(1.19–3.01)**	**0.007**	**1.62** **(1.01–2.59)**	**0.047**
Meet 3 guidelines	36 (9.4)	25 (8.3)	1.67 (0.89–3.15)	0.113	1.24 (0.66–2.34)	0.502

Bold values denote statistical significance at the *p* ≤ 0.05 level.

**Table 3:** Bivariant analysis in adolescent girls and boys.

Variable	Mode of transport		Bivariate analysis	
	AC *n* (%) *N* = 174	PC *n* (%) *N* = 174	OR 95% CI	*P*-value
Girls				
24-hour movement guidelines				0.099
Meet 0 guidelines	28 (16.1)	45 (25.9)	1	
Meet 1 guideline	76 (43.7)	76 (43.7)	1.61 (0.91–2.84)	0.102
Meet 2 guidelines	50 (28.7)	39 (22.4)	**2.06** **(1.10–3.87)**	**0.025**
Meet 3 guidelines	20 (11.5)	14 (8)	**2.30** **(1.00–5.27)**	**0.050**
Screen time ≤120 min/day				
No	138 (79.3)	146 (83.9)	1	0.269
Yes	36 (20.7)	28 (16.1)	1.36 (0.79–2.35)	
PA ≥60 min/day				0.238
No	76 (43.7)	87 (50)	1	
Yes	98 (56.3)	87 (50)	1.29 (0.85–1.97)	
Sleep time ≥8 h				**0.025**
No	72 (41.4)	93 (53.4)	**1**	
Yes	102 (58.6)	81 (46.6)	**1.62** **(1.06–2.48)**	
Boys				
24-hour movement guidelines				0.595
Meet 0 guidelines	22 (10.5)	12 (9.4)	1	
Meet 1 guideline	79 (37.8)	57 (44.5)	0.76 (0.35–1.65)	0.483
Meet 2 guidelines	92 (44)	48 (37.5)	1.05 (0.48–2.29)	0.912
Meet 3 guidelines	16 (7.7)	11 (8.6)	0.79 (0.28–2.25)	0.663
Screen time ≤120 min/day				0.485
No	179 (85.6)	106 (82.8)	1	
Yes	30 (14.4)	22 (17.2)	0.80 (0.44–1.47)	
PA ≥60 min/day				0.085
No	52 (24.9)	43 (33.6)	1	
Yes	157 (75.1)	85 (66.4)	1.53 (0.94–2.47)	
Sleep time ≥8 h				0.664
No	85 (40.7)	49 (38.3)	1	
Yes	124 (59.3)	79 (61.7)	0.90 (0.58–1.42)	

Bold values denote statistical significance at the *p* ≤ 0.05 level.

## 4 Discussion

Our results indicate that the prevalence of Spanish adolescents meeting all the 24-Hour Movement Guidelines was low but there was a relationship with the mode of commuting to school and gender. These findings showed a positive association between higher achievement of the 24-Hour Movement Guidelines and higher OR of ACS in adolescent girls.

The prevalence of adolescents meeting all the 24-hour Movement Guidelines is similar to a previous study (9). The low prevalence in our study in meeting the 24-Hour Movement Guidelines was mainly due to the low prevalence in meeting the ST recommendation, which was the same reason for UK youth (37). A cause for concern is the percentage of adolescents meeting no guidelines being higher (15.7%) than those that achieve all the 24-Hour Movement Guidelines (8.9%). Other studies show that a favorable health status is associated with meeting at least two movement behavior recommendations rather than with meeting a single recommendation (5, 10). For this reason, it is important to promote these recommendations together and WHO is already considering incorporating these recommendations in the GAPPA plan (6).

In our study, meeting at least two 24-Hour Movement Guidelines compare to meeting no guideline was significantly associated with ACS. Notably, AC were both more active and engaged in less ST than PC – and these differences were not at the expense of sleep given no differences in sleep between ACS and PC. This is the first study relating directly ACS and 24-Hour Movement Guidelines and, considering our results, we propose to include the mode of commuting to school as part of the 24-Hour Movement Guidelines, becouse ACS is a source of PA that contributes to meeting the Guidelines.

In our study we found that meeting two or three 24-Hour Movement Guidelines were associated with higher odds of ACS in girls. Due to the scientific evidence regarding the low levels of PA guidelines achievement in girls (38), it seems that the promotion of ACS could be one strategy to improve PA levels in girls together with 24-Hour Movement Guidelines. In addition, this is another reason to incorporate ACS into the 24-Hour Movement Guidelines.

Considering the cumulative health benefits of meeting the 24-Hour Movement Guidelines, our study can provide insightful information for policy-makers and practitioners for developing programs that will improve movement behaviors in a young population. Therefore, our investigation is helpful because it has shown that ACS is related to meeting more 24-Hour Movement Guidelines recommendations. It highlights ACS as a source of physical activity and the value of promoting ACS to improve healthy behaviors in adolescents.

Moreover, we need to bear in mind, that ACS is related to physical and social environmental determinants (e.g., urban design, safety, parental restrictions) (39), which may affect the relationship between ACS and 24-Hour Movement Guidelines. However, we feel that promoting ACS and its physical and social environmental determinants could be a good strategy to be addressed by local community, policy-makers and practitioners.

The strengths of our study include the following: our study was the first to examine the association between meeting 24-Hour Movement Guidelines and ACS in adolescents and, thus, can inform the design of effective interventions, prevention strategies, and policies; second, we included additional analyses by gender, related to the relationship between meeting the 24-Hour Movement Guidelines and ACS, which could help researchers to better understand the patterns of movement behaviors by gender.

This study included, however, some limitations that should also be mentioned. First, the cross-sectional study design precludes conclusions regarding causality between behaviors and ACS; second, a self-reported questionnaire was used, therefore, the PA measure was not an objective method. However, this measure was focus in understanding and predicting PA behavior change. Additionally, summing ST across all devices assumes no overlap in usage, which may inflate total ST. Future research using accelerometers is encouraged.

## 5 Conclusions

In conclusion, our study suggests that meeting more 24-Hour Movement Guidelines is associated with ACS in adolescents. In addition, promoting ACS as a strategy to meet the 24-Hour Movement Guidelines is recommended particularly for adolescent girls.

## Data Availability

The raw data supporting the conclusions of this article will be made available by the authors, without undue reservation.
